# Epidemiologic, Entomologic, and Virologic Factors of the 2014–15 Ross River Virus Outbreak, Queensland, Australia

**DOI:** 10.3201/eid2512.181810

**Published:** 2019-12

**Authors:** Cassie C. Jansen, Martin A. Shivas, Fiona J. May, Alyssa T. Pyke, Michael B. Onn, Kerryn Lodo, Sonja Hall-Mendelin, Jamie L. McMahon, Brian L. Montgomery, Jonathan M. Darbro, Stephen L. Doggett, Andrew F. van den Hurk

**Affiliations:** Communicable Diseases Branch, Queensland Government Department of Health, Herston, Queensland, Australia (C.C. Jansen, K. Lodo);; Brisbane City Council, Fortitude Valley, Queensland, Australia (M.A. Shivas, M.B. Onn);; Metro North Hospital and Health Service, Windsor, Queensland, Australia (F.J. May);; Forensic and Scientific Services, Queensland Government Department of Health, Coopers Plains, Queensland, Australia (A.T. Pyke, S. Hall-Mendelin, J.L. McMahon, A.F. van den Hurk);; Metro South Hospital and Health Service, Coopers Plains (B.L. Montgomery);; Queensland Institute of Medical Research Berghofer, Herston (J.M. Darbro);; University of Sydney and Westmead Hospital, Sydney, New South Wales, Australia (S.L. Doggett)

**Keywords:** Ross River virus, epidemic polyarthritis, outbreak, mosquitoes, Brisbane, Queensland, Australia, epidemiology, entomology, viruses, vector-borne infections, *Culex annulirostris*, *Aedes procax*, *Culex orbostiensis*, *Mansonia uniformis*, sequence analysis, zoonoses

## Abstract

Australia experienced its largest recorded outbreak of Ross River virus (RRV) during the 2014–15 reporting year, comprising >10,000 reported cases. We investigated epidemiologic, entomologic, and virologic factors that potentially contributed to the scale of the outbreak in Queensland, the state with the highest number of notifications (6,371). Spatial analysis of human cases showed that notifications were geographically widespread. In Brisbane, human case notifications and virus detections in mosquitoes occurred across inland and coastal locations. Viral sequence data demonstrated 2 RRV lineages (northeastern genotypes I and II) were circulating, and a new strain containing 3 unique amino acid changes in the envelope 2 protein was identified. Longitudinal mosquito collections demonstrated unusually high relative abundance of *Culex annulirostris* and *Aedes procax* mosquitoes, attributable to extensive freshwater larval habitats caused by early and persistent rainfall during the reporting year. Increased prevalence of these mosquitoes probably contributed to the scale of this outbreak.

Ross River virus (RRV; family *Togaviridae*, genus *Alphavirus*) is distributed throughout Australasia and has caused outbreaks involving thousands of humans in the western Pacific (*1*). RRV is the most commonly reported endemic arboviral infection in Australia; a mean of 4,541 cases/year were recorded during 2000–2016 (*2*). Cases are reported from every state and territory of Australia, and Queensland accounts for a large percentage (40%–65% during 2000–2006) (*2*).

Similar to the disease spectrum of related chikungunya virus, RRV infection causes polyarthritis and, in some cases, fever, maculopapular rash, fatigue, myalgia, lethargy, and headache (*3*,*4*). Many infections are asymptomatic and do not result in clinical disease (5), but debilitating arthritis of 3–6 months’ duration can occur in some patients (*5*–*7*). RRV ecology is complex, involving zoonotic transmission between multiple mosquitoes and vertebrates (*8*). Although numerous species may be hosts for RRV, the predominant vertebrate hosts are considered to be macropods (e.g., kangaroos and wallabies) (*1*,*9*,*10*). Humans have been implicated as hosts in outbreaks where macropods were absent (*11*,*12*). Overall, >40 mosquito species have yielded RRV isolates, although *Aedes vigilax*, *Aedes* *camptorhynchus*, and *Culex annulirostris* mosquitoes are considered the key vectors (*13*). Other species can be involved in specific locations (*8*,*14*), and transmission dynamics appear locally unique.

During the 2014–15 reporting year (i.e., July 1, 2014–June 30, 2015), a widespread RRV epidemic occurred in Australia; 10,074 cases were reported to the National Notifiable Diseases Surveillance System (*15*). This epidemic represented the highest number of RRV notifications ever reported in a season since 1993, when human RRV infection became nationally notifiable. In total, 63% (6,371) of notifications were from Queensland, Australia’s third-most populous state (*15*). We investigated the epidemiologic, entomologic, and virologic characteristics of the outbreak in Brisbane, the Queensland capital.

## Methods

### Study Area

Brisbane is situated at 27°28′S and 153°01′E on Australia’s eastern coast. The Brisbane local government area (LGA) comprises 1,367 km^2^ and, on June 30, 2015, had an estimated residential population of 1,165,437 (*16*). Brisbane has a subtropical climate (Köppen climate classification Cfa); monthly average temperatures are 10°C–22°C in winter and 20°C–29°C in summer. Approximately two thirds of the annual mean rainfall (1,149 mm) falls during November–March (*17*).

### Human Case Notifications

The Queensland Notifiable Conditions Surveillance System (*18*) houses data on notifiable conditions in Queensland as outlined in the Public Health Act 2005 (*19*). We defined an RRV notification as the national case definition (i.e., a laboratory diagnosis of RRV) (*20*), but in 2016, this definition was changed to reduce the effect of false-positive notifications resulting from single IgM-positive test results. Thus, notifications reported herein might include false-positives. We assigned an LGA to notified cases using patient residential addresses. We extracted notification data, including date of specimen collection (used as a proxy for illness onset because this information was not systematically collected), residential address, and LGA, from the Notifiable Conditions Surveillance System for the period January 1, 1990–June 30, 2015. We present data as annual totals by reporting year, defined as July 1 of one year through June 30 of the next year, to reflect seasonality of mosquito abundance and mosquitoborne disease notifications and provide consistency with the national reporting convention. We numbered weeks as specified by ISO 8601:2004 (*21*), with week 1 starting on a Monday and containing the first Thursday of the calendar year.

We tabulated RRV notifications in the Brisbane LGA by week of specimen collection and Australian Statistical Geography Standard statistical area level 2 (*22*) and visualized using QGIS 2.18.1 (https://qgis.org). Because locations of exposures were unknown, we used patient residential address to map the spatial distribution of notifications. We performed all case data analyses in Stata SE 15 (https://www.stata.com) and calculated rates (per 100,000 population) using estimated Queensland residential population data (*23*). We obtained ethics approval to conduct this research through the Children’s Health Queensland Hospital and Health Service Human Research Ethics Committee (reference no. HREC/15/QRCH/230).

### Mosquito Collections

**Figure 1:**
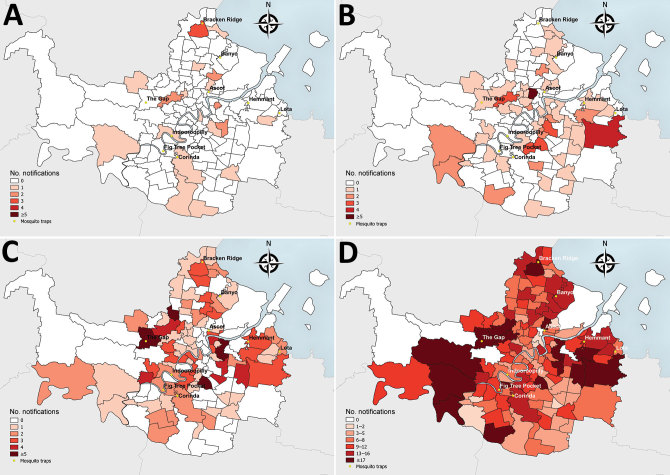
Spatial distribution of Ross River virus notifications by patient residential address and Australian Statistical Geography Standard statistical area level 2 (*22*) and mosquito trap sites, Brisbane local government area, Queensland, Australia, 2015. A) Week 2 (first week with an increased number of cases); B) week 6 (early in outbreak); C) week 9 (peak of notified cases); and D) weeks 2–20 combined (entire outbreak period).

We collected mosquitoes weekly at 9 sites representing the larval habitat diversity of implicated RRV vectors and their proximity to human habitation. Trap sites varied by distance to larval habitats. Four sites were within 500 m of a saltmarsh, and 5 were close to freshwater habitats; some freshwater habitats were also near urban areas and considered suburban larval habitats (Table 1; Figure 1). We collected mosquitoes using PB light traps (Pacific BioLogics, http://www.pacificbiologics.com.au) baited with carbon dioxide (2-kg dry ice pellets) and 1-octen-3-ol (*24*) operated 4:00 PM–7:00 AM.

**Table 1:** Mosquito trap site location and type, Brisbane local government area, Queensland, Australia

Site name, suburb	Geolocation	Dominant habitat type
Ascot	–27.431441, 153.051788	Suburban hilltop, freshwater
Bracken Ridge	–27.307225, 153.040433	Saltmarsh
Banyo	–27.369166, 153.072694	Saltmarsh
Corinda	–27.549861, 152.994836	Suburban, freshwater
Hemmant	–27.451706, 153.123781	Saltmarsh
Indooroopilly	–27.511639, 152.984458	Suburban riparian
Lota	–27.469912, 153.18057	Saltmarsh
The Gap	–27.450889, 152.937806	Suburban, freshwater
Fig Tree Pocket	–27.539056, 152.969333	Suburban, freshwater

To account for occasional variation in the number of traps set (resulting from trap failures and prohibitive weather), for each week, we calculated the mean count of all mosquito species per trap and mean relative abundance of each mosquito species per trap. We compared the mean count of all mosquitoes per trap in the 2014–15 season with those of the other reporting years using Poisson regression. We compared the mean relative abundance of mosquito species comprising >5% of the total trap catch in 2014–15 with their mean relative abundances in the previous 2 reporting years using the 2-sample test of proportions. We considered p values <0.05 significant for all statistical tests.

For each week, we compared the number of human cases notified in the Brisbane LGA with the mean total mosquito count per trap and the mean relative abundance of frequently collected mosquitoes (i.e., those comprising >5% of the total trap catch in 2014–15) using Spearman rank correlation. We similarly compared the lag time of 0–8 weeks between mosquito counts and human case notifications.

### Virus Detection in Mosquito Saliva and Mosquito Pools

We used 2 methods to acquire mosquito samples for RRV screening (Appendix). The first method was the sugar-based system described by Flies et al. (*25*), which involves collecting mosquito saliva expectorated during feedings (*26*). We deployed traps containing honey-soaked Flinders Technology Associates (FTA) cards (Whatman International Ltd, https://www.gelifesciences.com) overnight on 15 occasions at weekly intervals during February 3–May 20, 2015 (weeks 6–21), excluding week 18. For the second method, we pooled whole mosquitoes collected in traps during February 3–March 10, 2015 (weeks 6–11), by species, trap, and trap night into groups of <100 mosquitoes.

We used a cell culture ELISA (*27*) to detect RRV in mosquito pools. We used an RRV-specific TaqMan real-time reverse transcription PCR (rRT-PCR) (*28*) to detect RRV RNA extracted from FTA cards and RRV RNA from mosquito pools acquired from traps that yielded RRV-positive FTA cards. We also performed rRT-PCR on mosquito samples derived from traps where a high level of mosquito death was observed during the 24-hour holding period after trap collection. Mosquito death compromises virus integrity and subsequent detection in the cell culture ELISA.

### Sequence Analysis

We extracted virus RNA from patient serum samples, mosquito homogenates, FTA cards, and infected C6/36 cell culture supernatants. We amplified and sequenced the complete envelope (E) 3 and E2 gene regions (1,458 nt in total) using RRV-specific primers (Appendix Table 2) and 2 overlapping RT-PCR reactions (Appendix). We phylogenetically compared the RRV E3 and E2 sequences from samples collected in Brisbane during the 2014–15 outbreak with those of archived viruses from Brisbane and other locations around Australia isolated during 1959–2016 (Appendix Table 1).

**Table 2:** Total rainfall in Brisbane local government area, Queensland, Australia, 2011–2015, compared with long-term average

Reporting year	Rainfall, mm*	% Long-term average rainfall†
2011–12	1,305	124
2012–13	1,159	110
2013–14	582	55
2014–15	1,595	152

*Rainfall values from the Bureau of Meteorology (*17*). †The long-term average rainfall for the reporting years 2000–2015 was 1,049 mm.

## Results

### Study Area Climate

The weather of southeast Queensland during the 2014–15 reporting year was characterized by early and consistent weekly rainfall from mid-November through late February (*17*), followed by drier weather interspersed with several large rain events. A total of 1,595 mm of rain fell, representing 152% of the Brisbane long-term average (Table 2). Of note, the preceding reporting year was unusually dry; only 55% of the long-term average rainfall fell.

### Human Case Notifications

**Figure 2:**
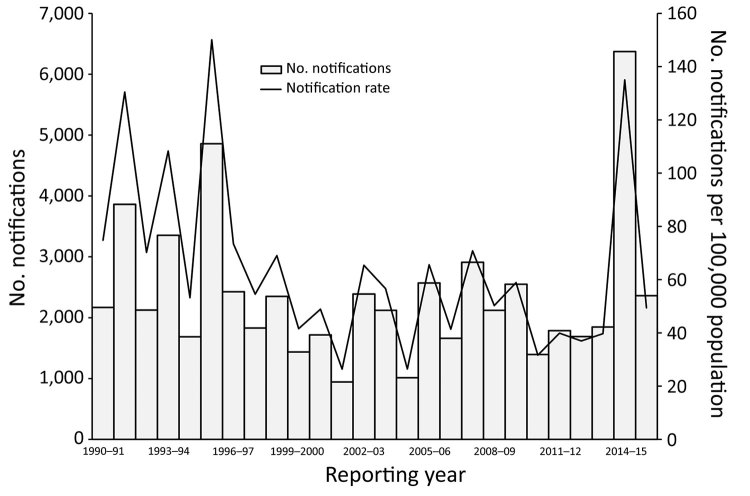
Number of notifications and notification rate of Ross River virus infections by reporting year, Queensland, Australia, 1990–2016. Reporting year is defined as July 1 of one year to June 30 of the next year.

In the 2014–15 reporting year, 10,074 RRV notifications were reported nationally through the National Notifiable Diseases Surveillance System. The number of notifications in Queensland was 6,371, considerably higher than the mean of 1,854 cases reported annually over the previous 5 years and the largest number reported since statewide RRV surveillance began in 1990. Despite being the highest number of annual RRV notifications reported, the Queensland notification rate in 2014–15 (135 notifications/100,000 population) was lower than that in 1995–96 (150 notifications/100,000 population; Figure 2), a finding attributable to an increase in population over time. However, the mean rate for the 5 years before the 2014–15 outbreak was 41 notifications/100,000 population.

**Figure 3:**
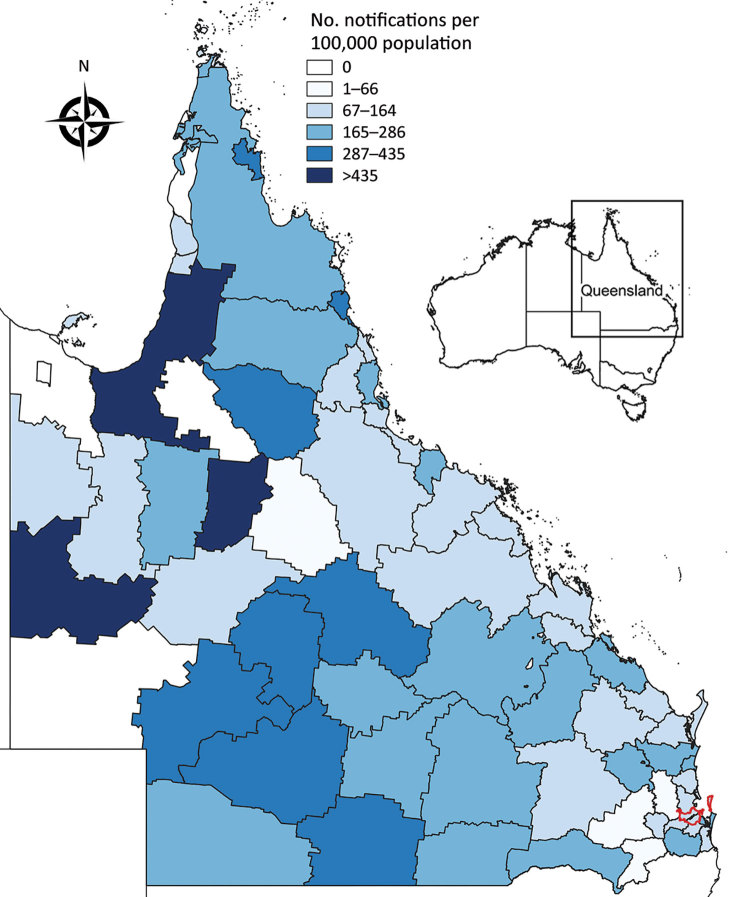
Ross River virus notification rate by local government area, Queensland, Australia, July 1, 2014–June 30, 2015. Brisbane local government area (red outline) is indicated. Inset map shows the location of Queensland in Australia.

**Figure 4:**
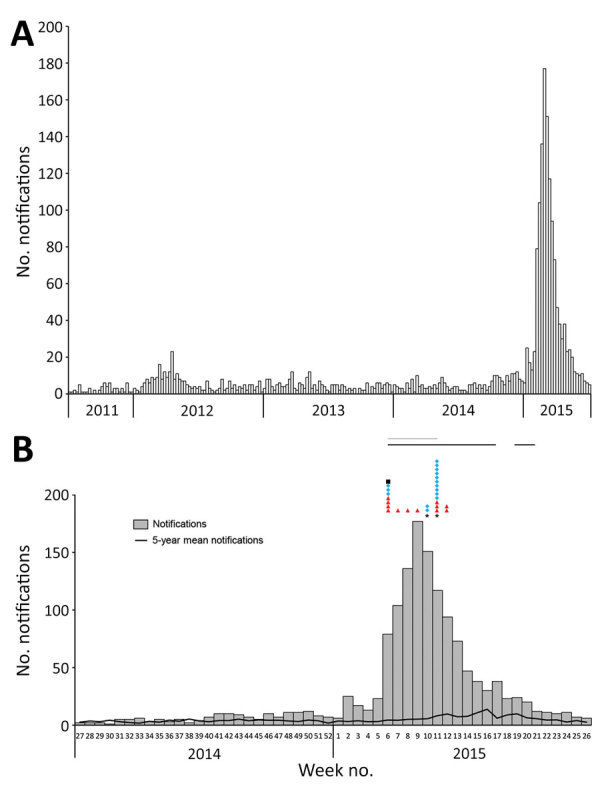
Ross River virus (RRV) notifications by week, Brisbane local government area, Queensland, Australia, July 1, 2011–June 30, 2015 (A), and July 1, 2014–June 30, 2015 (B). Symbols in panel B represent single detection events: red triangles, RRV RNA detection from Flinders Technology Associates cards by real-time reverse transcription PCR; blue diamonds, RRV RNA detection from mosquito pools by real-time reverse transcription PCR; and black square, RRV detection from mosquito pools by cell culture ELISA. Also in panel B, the black line above the graph indicates when Flinders Technology Associates cards were deployed and gray line when mosquito pools were being collected and screened for RRV infection. Mosquitoes acquired from traps in weeks 10 and 11 were damaged by rain; thus, RRV-positive mosquito parts might have stuck to RRV-negative mosquitoes and turned some pools artificially positive.

The 2014–15 notification rates varied by Queensland LGA (Figure 3). In total, 1,454 RRV notifications were reported in the Brisbane LGA in 2014–15. The number of weekly notifications first increased in Brisbane in week 2 of 2015 (25 cases; Figure 1, panel A; Figure 4). A marked increase occurred in week 6 (79 cases, compared with the average of 16.8 cases of the preceding 5 weeks), and the highest number occurred in week 9 (177 cases; Figure 1, panels B, C; Figure 4). The number of weekly case notifications returned to pre-outbreak levels by week 21 (Figure 4).

Notifications were widespread across Brisbane throughout the outbreak (Figure 1, panels A–D). No spatial clustering by statistical area level 2 was observed for notifications at any time during the outbreak.

### Mosquito Collections

During 2014–15, a total of 411,328 mosquitoes (mean 877 mosquitoes per trap night) comprising >35 species were collected (Appendix Table 3). This number is a significant increase compared with the 204,220 (mean 498 mosquitoes/trap night) collected during the 2012–13 reporting year and 108,422 (mean 232 mosquitoes/trap night) collected during the 2013–14 reporting year (p<0.001). *Ae*. *vigilax*, *Cx. annulirostris*, and *Aedes procax* mosquitoes were the only species that comprised >5% of the trap catch during the 2014–15 reporting year. *Ae. vigilax* populations dominated collections in all years. Only *Cx. annulirostris* and *Ae*. *procax* mosquitoes significantly increased in abundance during the 2014–15 reporting year compared with previous reporting years (p<0.001). The relative abundance of all other species was not significantly increased in 2014–15 compared with previous years.

*Cx. annulirostris* populations accounted for 34% (140,287/411,328) of the total trap catch in 2014–15, a relative abundance significantly higher than those recorded for the 2012–13 (20%, 39,858/204,220; p<0.001) and 2013–14 (12%, 12,650/108,422; p<0.001) reporting years. During 2014–15, *Cx. annulirostris* mosquitoes showed an earlier than usual increase in abundance, and elevated counts were sustained throughout the outbreak (data not shown). The initial increase in weekly collections of this mosquito population observed starting week 50 of 2014 coincided with an increased number of weekly case notifications. The correlation between the mean relative abundance of *Cx. annulirostris* populations and RRV notifications was strong and significant (Spearman rank correlation coefficient ρ = 0.6190; p<0.001) only when a 3-week lag from mosquito abundance to human case notifications was applied. No correlation between the mean relative abundance of *Cx. annulirostris* mosquitoes and RRV notifications was observed in other reporting years (data not shown).

The *Ae*. *procax* population accounted for 6.4% (26,408/411,328) of the total trap catch in 2014–15, a relative abundance significantly higher than those recorded for the 2012–13 (2.3%, 4,654/204,220; p<0.001) and 2013–14 (1.4%, 1,570/108,422; p<0.001) reporting years. As with *Cx. annulirostris* mosquitoes, *Ae*. *procax* mosquito abundance increased starting week 50 of 2014 but did not reach a sustained peak until week 10 of 2015 and did not decrease until week 18 of 2015 (data not shown). As a result, *Ae*. *procax* mosquito mean relative abundance only moderately correlated with RRV notifications; a 2-week lag produced the highest correlation (ρ = 0.5543; p<0.001). No correlation was observed in any other reporting year (data not shown).

More *Ae*. *vigilax* mosquitoes were collected in 2014–15 than in other years. However, the relative abundance was only 51% (211,008/411,328) of the total trap catch, significantly lower than that of 2012–13 (60%, 123,024/204,220; p<0.001) and 2013–14 (78%, 84,133/108,422; p<0.001). *Ae*. *vigilax* mosquito numbers peaked in December 2014 (data not shown) but returned to typical numbers by early January, consistent with a weak negative correlation with RRV notifications (ρ = –0.3553, p = 0.009).

### Virus Detection in Mosquito Pools and FTA Cards

A total of 135 honey-soaked FTA cards were deployed in mosquito traps during February 3–May 20, 2015, and we detected RRV RNA on 12 (8.9%) of them (Figure 4, panel B). On the first week of deployment (week 6 of 2015), 4 cards were positive for RRV RNA. Except for week 10, >1 card was positive each week during weeks 6–12, after which RRV was not detected. RRV was detected 3 times from FTA cards deployed March 11, 2015 (week 11), and 2 times from cards deployed March 18, 2015 (week 12; Figure 4, panel B). Except for Fig Tree Pocket, RRV RNA was detected >1 time from each trap location.

We processed 21,250 mosquitoes (5% of total collected in 2014–15), representing >20 species, for RRV detection (Appendix Table 4). Mosquitoes were combined into 385 pools and screened by cell culture ELISA. We also processed 155 pools, representing 10,112 mosquitoes, for rRT-PCR. A single pool of 68 *Cx. annulirostris* mosquitoes collected from Lota in week 6 of 2015 was positive by cell culture ELISA and rRT-PCR. One pool each of *Ae. vigilax* and *Culex orbostiensis* mosquitoes collected in the same trap on the same trap night as the *Cx. annulirostris* population were positive by rRT-PCR. RRV was also detected in a pool of 4 *Mansonia uniformis* mosquitoes collected in week 10 of 2015 at Hemmant. Viral RNA was detected in an additional 11 pools comprising mosquitoes from the trap deployed at Hemmant in weeks 10 (1 pool) and 11 (10 pools) of 2015. However, the species of mosquitoes in these pools could not be identified morphologically because of rain permeated the traps and damaged the samples. Thus, the high number of RRV-positive pools from these traps could represent cross-contamination caused by parts of RRV-positive mosquitoes sticking to RRV-negative mosquitoes. Regardless, these data are evidence that RRV was present at Hemmant during these weeks.

### Virus Nucleotide Sequence Phylogenetic Analysis

**Figure 5:**
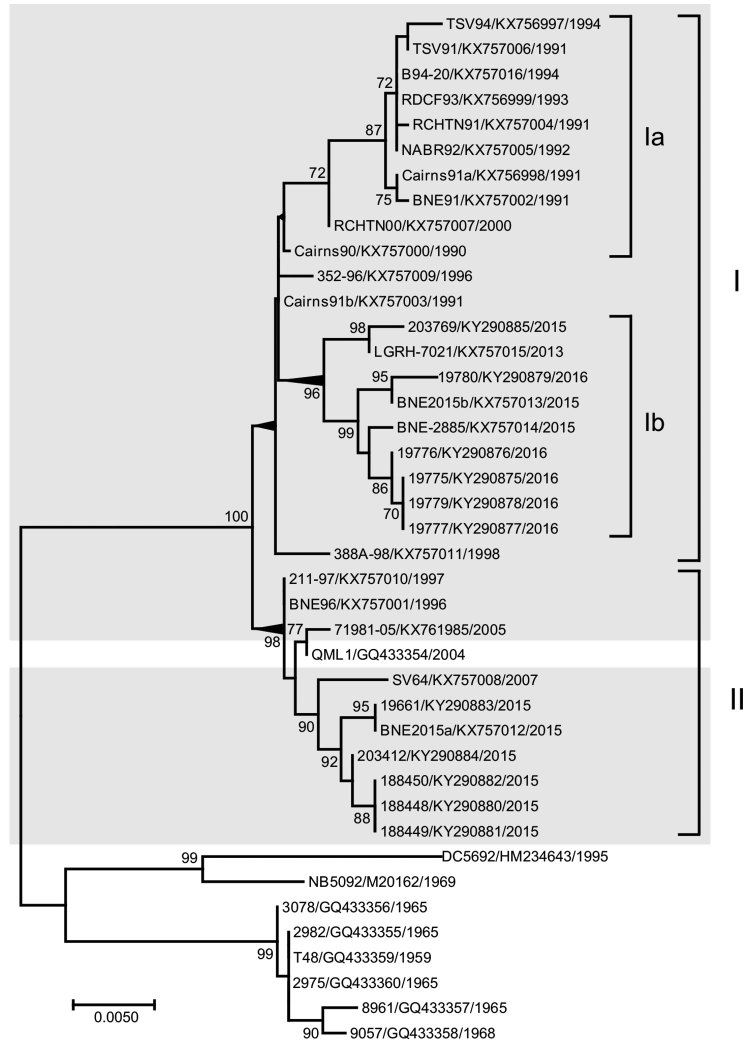
Maximum-likelihood phylogenetic tree of 41 complete Ross River virus envelope (E) 3 and E2 gene nucleotide sequences (1,458 nt), 32 from isolates collected in Queensland and New South Wales, Australia, during January 1, 1990–June 30, 2015 (gray shading), and 9 reference sequences. Tree was constructed by using MEGA 7.0 (https://megasoftware.net) with bootstrap support (1,000 replications). The tree is midpoint rooted for clarity. Circulating northeastern lineages I and II are shown together with sublineages Ia and Ib. Percentage bootstrap support values determined from 1,000 replicates are shown for key nodes. GenBank accession numbers are provided. Scale bar indicates nucleotide substitutions per site.

We determined the complete E3 and E2 gene sequences of 32 RRV samples and phylogenetically compared them with 9 additional RRV sequences from GenBank (accession nos. HM234643, M20162, GQ433354–60). The maximum-likelihood phylogenetic tree inferred from these sequences demonstrated all isolates belonged to the northeastern genotype (Figure 5). The 32 RRV sequences sampled over a 27-year period grouped within 1 of 2 major northeastern lineages, designated I and II (Figure 5). The phylogenetic groupings of BNE2015b (human origin, GenBank accession no. KX757013) and BNE-2885 (mosquito origin, GenBank accession no. KX757014) from Brisbane into lineage I and BNE2015a (human origin, accession no. KX757012) from Brisbane and 19661 (mosquito origin, accession no. KY290883) from Tweed, New South Wales, Australia, into lineage II demonstrate co-circulation of both lineages in southeast Queensland and northeast New South Wales during the 2014–15 outbreak.

Sequences of the outbreak isolates BNE2015b (lineage I) and BNE2015a (lineage II) were highly similar (98.4% nucleotide identity, 99.0% amino acid identity). Within lineage I, 2 sublineages (Ia and Ib) were demonstrated (Figure 5). In a comparison of amino acid sequences, except for the 203769 isolate (Queensland 2015) sequence, which was most similar to the LGRH-7021 (Longreach, Queensland, 2013) isolate sequence, the 2015 and 2016 sublineage Ib sequences all contained an A389T substitution within E2. Within lineage II, the E3 and E2 sequences of isolates 19661 (from an FTA card) and BNE2015a (from a patient) sampled during the 2014–15 outbreak shared 100% nucleotide and amino acid identities. Of note, these 2 sequences contained 3 unique amino acid substitutions in the E2 gene (A369T, M376I, T384A). Another unique E2 amino acid substitution, M45K, was demonstrated in 3 New South Wales 2015 RRV sequences obtained from mosquitoes (188448–50).

## Discussion

Outbreaks of RRV involving hundreds to thousands of cases have been reported from all mainland states of Australia (*29*). The 2014–15 outbreak was unprecedented in the high number of cases reported and large area of the eastern seaboard affected. Our investigations confirmed that human case notifications were distributed across the Brisbane LGA throughout the season, including before the outbreak, early in the outbreak, and at the peak of notifications. The concurrent detection of virus from mosquitoes across Brisbane provides compelling evidence that RRV activity was widespread and the exposure risk for humans high across all suburbs and districts. We suggest that a combination of ecologic factors contributed to the magnitude of the RRV outbreak in Brisbane in 2014–15.

Previous RRV outbreaks in Australia were preceded by above-average rainfall (*29*,*30*). The weather in Brisbane during 2014–15 was unusual, characterized by early elevated rainfall that persisted throughout the summer and resulted in total rainfall exceeding the historical mean. These conditions provided temporary freshwater larval habitats for many mosquito species, including *Cx. annulirostris* populations, for an unusually long period. The early increase in *Cx. annulirostris* abundance, which remained high, coupled with a correlation with RRV notifications, suggest that this species was a key vector during the outbreak. In addition, the widespread geographic distribution of *Cx. annulirostris* mosquitoes (data not shown), which reflected the distribution of human notifications, further supports the involvement of this species in the outbreak. The *Cx. annulirostris* mosquito is a competent laboratory vector of RRV that has yielded numerous field isolates in previous studies (*31*,*32*) and yielded field isolates in our study. Furthermore, evidence has implicated *Cx. annulirostris* mosquito involvement in RRV outbreaks in New South Wales in 2014–15 (*33*) and New South Wales and Victoria in 2016–17 (*34*,*35*).

On the basis of their temporal and spatial abundance, *Ae*. *procax* mosquitoes also showed a moderate correlation with human RRV notifications in 2014–15, albeit at a lower relative abundance than *Cx. annulirostris* mosquitoes. Although RRV was not detected in the *Ae*. *procax* populations herein, this species has previously yielded relatively high numbers of field isolates (when compared with the number of specimens tested) and demonstrates high vector competence for RRV in the laboratory (*32*). Like *Cx. annulirostris* mosquitoes*, Ae*. *procax* mosquitoes feed on a range of mammals (*36*), so they might play a greater role in urban transmission of arboviruses than previously considered (*32*,*37*,*38*).

The most abundant saltmarsh mosquito in southeast Queensland, *Ae. vigilax*, reached notably high numbers in 2014–15. However, this mosquito’s relative abundance was significantly lower in 2014–15 than in previous years. Furthermore, the temporal abundance of *Ae. vigilax* populations peaked earlier and had a weak and negative correlation with human case notifications, suggesting that even if involved in enzootic transmission this species was unlikely responsible for sustained transmission to humans throughout the outbreak. In addition, in previous years, high numbers of *Ae. vigilax* mosquitoes were present in the Brisbane LGA without increased numbers of RRV notifications (e.g., 2012–13 and 2013–14), and low numbers were present in years when RRV notifications were above average (e.g., 2011–12).

Given the complexity of RRV transmission cycles, the role of other common species should not be discounted. Of the remaining 2 species from which RRV was detected during this study, *Ma. uniformis* mosquitoes have previously yielded isolates and been shown to transmit the virus in laboratory experiments (*32*). In contrast, RRV has not been detected in *Cx. orbostiensis* mosquitoes previously, despite extensive testing for field isolates in New South Wales since 1988, so its status as an RRV vector is unknown.

In Australia, RRV comprises 3 distinct genotypes, western, northeastern, and southeastern, named for the location in which they predominate (*39*,*40*). The finding of northeastern genotype lineage I and II sequences in human and mosquito samples suggests both lineages contributed to the 2014–15 outbreak and confirms their persistent transmission in eastern Australia. Our results are consistent with previous studies suggesting that the distribution of lineages I and II in eastern Australia are not constrained by geographic distance or location.

We detected several amino acid substitutions in E2 of most 2015 and 2016 RRV isolates, including 3 (A369T, M376I, T384A) in a strain represented by isolates 19661 and BNE2015a. Of note, A369T, M376I, T384A, and A389T all occurred within the putative E2 C-terminal anchor sequence comprising amino acids 365–90 (*41*). Whether these amino acid changes are pleiotropic or represent adaptive changes related to the interaction of E2 with E1 or other structural proteins during viral assembly is unknown.

We investigated entomologic, epidemiologic, and virologic factors associated with the 2014–15 RRV outbreak in Brisbane. A missing factor in the investigation of this and previous outbreaks is the contribution of nonhuman hosts to epidemic transmission. Numerous vertebrate species are likely involved in RRV maintenance (*10*), and the role of each species during outbreaks is probably complex. The widespread distribution of RRV during 2014–15 suggests the involvement of a common ubiquitous species or several reservoir species. Furthermore, limited RRV activity in the preceding years might have increased the pool of nonimmune hosts, contributing to the scale of the outbreak.

Overall, early and consistent rainfall in 2014–15 in southeast Queensland probably contributed to a high abundance and the survival of adult mosquitoes, providing ideal conditions for the largest recorded outbreak of RRV. As demonstrated by the spatial distribution of RRV patients and virus detections in mosquitoes, virus activity was widespread across the Brisbane LGA. Notwithstanding the potential role of other mosquito species in ongoing transmission of RRV, we propose that freshwater species (particularly *Cx. annulirostris* and *Ae*. *procax* mosquitoes) were likely key drivers of the outbreak activity in Brisbane in 2014–15. We demonstrate that the risk for RRV infection in humans is widespread and driven by complex factors in Queensland.

Appendix Additional information on the epidemiologic, entomologic, and virologic factors of the 2014–15 Ross River virus outbreak, Queensland, Australia.
