# Supplementary material for: Epidemiologic, Entomologic, and Virologic Factors of the 2014–15 Ross River Virus Outbreak, Queensland, Australia
Source: Emerg Infect Dis. 2019 Dec;25(12):2243–52. doi: 10.3201/eid2512.181810 (PMC6874252; doi:10.3201/eid2512.181810)
Supplement: Appendix — Additional information on the epidemiologic, entomologic, and virologic factors of the 2014–15 Ross River virus outbreak, Queensland, Australia. [file 18-1810-Techapp-s1.pdf]

Article DOI: https://doi.org/10.3201/eid2512.181810

Epidemiologic, Entomologic, and Virologic Factors of the 2014–15 Ross River Virus Outbreak, Queensland, Australia

Appendix

Methods

Virus Detection in Mosquitoes

Sugar feeding stations were prepared by coating Flinders Technology Associates (FTA) cards (Whatman International Ltd, Maidstone, UK) with a mixture of honey (Capilano, Richlands, Australia) and blue food dye (Queen Fine Foods, Alderley, Australia). The sugar mixture was left to soak into the cards for ≈4 hr before the card was inserted into a vinyl sleeve with a circular 'window' cut into 1 side to permit access by mosquitoes to feed. The completed sugar feeding station was suspended from a hook inside the mosquito collection chamber attached to each light trap. Upon collection of the traps from the field, the plastic collection chambers containing the mosquitoes were removed and placed in a large plastic box containing damp towels and stored at room temperature. Approximately 24 hr after collection, the collection chambers were removed from the larger box and mosquitoes were killed by exposure to CO_2 gas. The FTA cards were removed from the trap collection chambers and transported within 24 h to Forensic and Scientific Service, Brisbane, for analysis.

Mosquitoes to be submitted for virus detection were immediately frozen at –20°C following exposure to CO_2, morphologically identified using the taxonomic keys of Marks (*1*) and Lee et al. (*2*), and sorted by species into pools of ≤100 on a refrigerated table, before storage at –80°C for virus detection. Mosquitoes that were not sorted immediately were transferred to –80°C to await identification and pooling for virus detection.

Mosquito pools were transferred to 2 mL or 5 mL U-bottom tubes for pool sizes of ≤30 or >30 mosquitoes, respectively. A single 5-mm stainless steel ball and either 1.5 mL or 4 mL of growth media (Opti-MEM [GIBCO, Invitrogen Corporation, Grand Island, NY] containing 3%

fetal bovine serum, antibiotics, and antimycotics) was added to the 2 mL or 5 mL tubes, respectively. The pools were homogenized using either a Spex 8000 mixer/mill (Spex Industries, Edison, NJ) for pools of 31–100 mosquitoes or a QIAGEN TissueLyser II (QIAGEN, Hilden, Germany) for pools containing ≤30 individuals, before being clarified by centrifugation and filtration through a 0.8/0.2 µm dual filter (Pall Corporation, Ann Arbor, MI).

Filtered mosquito homogenates were inoculated onto *Aedes albopictus* (C6/36) cell monolayers in a 96-well microtiter plate. After a 7-d incubation at 28°C, cell monolayers were fixed in PBS/acetone. Virus antigen was detected using a cell culture enzyme immunoassay (*3*) and the monoclonal antibody B10. The FTA cards were processed as described previously (*4*). Nucleic acids were extracted from 140 µL of mosquito pool filtrate or FTA card eluates using the QIAGEN BioRobot Universal System and QIAamp Virus BioRobot MDx Kit (QIAGEN, Clifton Hill, Australia). A RRV-specific TaqMan real-time reverse transcription PCR (rRT-PCR) assay was used to detect RRV in extracted RNA and a positive result was recorded in the case of cycle threshold <40 (*5*).

Virus Strain Sequence Analysis

Samples used for the sequence analysis included patient serum, mosquito homogenate, FTA card, or infected C6/36 cell culture supernatant obtained from isolates (Appendix Table 1). Passage 1 was used for all isolates, with the exception of New South Wales mosquito isolates 188448–188450, 203412, and 203769, which were previously passaged twice in baby hamster kidney cells followed by 1 passage in C6/36 cells. Viral RNA was extracted using the QIAmp Viral RNA Extraction Kit (QIAGEN, Germany) according to the manufacturer's instructions. Amplification of complete envelope (E) 3 and E2 gene regions (1,458 nt) was performed using RRV-specific primers (Appendix Table 2) and 2 overlapping rRT-PCR reactions (reaction A primers 8183for and 9253rev and reaction B primers 8870for and 10298rev). For low level RNA samples, sensitivity was increased by further amplification of the rRT-PCR DNA products in 2 respective overlapping nested PCRs (reaction A primers 8313for and 9253rev and reaction B primers 8999for and 10245rev). For first round rRT-PCR amplification, the Superscript® III One-Step System with High Fidelity Platinum® *Taq* (Invitrogen Life Technologies, Thermofisher, Waltham, MA, USA) was used according to the manufacturer's instructions, with the exception that 20 µM of each primer, and 5 µL of sample RNA was used in the 50 µL reaction. The cycling conditions consisted of 1 cycle at 50°C for 15 min and 94°C for 2 min; 40

cycles at 94°C for 15 sec, 55°C for 30 sec, and 68°C for 1 min; and a final extension step at 68°C for 5 min. For the nested PCRs, DNA products from the rRT-PCR reactions were diluted 1:100 and amplification was performed using 5 µL of the diluted DNA in the MyFi DNA Polymerase System (Bioline, London, United Kingdom) according to the manufacturer's instructions with total reaction volume of 25 µL. The cycling conditions for the nested PCRs consisted of 1 cycle at 95°C for 1 min; 35 cycles at 95°C for 15 sec, 55°C for 30 sec, and 72°C for 1 min; and a final extension step at 72°C for 5 min. Nucleotide sequencing of the amplified E3 and E2 genes was performed with specific RRV oligonucleotide primers (Appendix Table 2) using the Big Dye® Terminator v3.1 cycle sequencing kit (Applied Biosystems, Thermofisher, USA) according to the supplier's protocols in the 3130 Genetic Analyzer (Applied Biosystems, USA).

Nucleotide sequences derived from RRV samples and isolates were deposited in GenBank (Appendix Table 1). Complete E3 and E2 gene nucleotide sequence (1,458 nt) alignments were performed using Clustal W and Mega 7.0 software (*6*). The GTR + G nucleotide substitution model was chosen following scrutiny with JModelTest 2 software (*7*). A maximum likelihood tree with bootstrap support (1,000 replicates) was inferred from 41 RRV sequences and nucleotide and amino acid pairwise distances were determined using the p-distance model in Mega 7.0.

Appendix References

1. Marks EN. An Atlas of Common Queensland Mosquitoes. Herston, Queensland: Queensland Institute of Medical Research; 1982.

2. Lee DJ, Hicks MM, Griffiths M, Debenham ML, Bryan JH, Russell RC, et al. The Culicidae of the Australasian region. Entomology Monograph No. 2. Volumes 1–12. Canberra: Australian Government Publishing Service Press; 1980–1989.

3. Broom AK, Hall RA, Johansen CA, Oliveira N, Howard MA, Lindsay MD, et al. Identification of Australian arboviruses in inoculated cell cultures using monoclonal antibodies in ELISA. Pathology. 1998;30:286–8. PubMed https://doi.org/10.1080/00313029800169456

4. Hall-Mendelin S, Ritchie SA, Johansen CA, Zborowski P, Cortis G, Dandridge S, et al. Exploiting mosquito sugar feeding to detect mosquito-borne pathogens. Proc Natl Acad Sci U S A. 2010;107:11255–9. PubMed https://doi.org/10.1073/pnas.1002040107

5. Hall RA, Prow NA, Pyke AT. Ross River virus. In: Liu D, editor. Molecular detection of human viral pathogens. Boca Raton, FL: CRC Press; 2011. p. 349–59.

6. Kumar S, Stecher G, Tamura K. MEGA7: Molecular Evolutionary Genetics Analysis version 7.0 for bigger datasets. Mol Biol Evol. 2016;33:1870–4. PubMed https://doi.org/10.1093/molbev/msw054

7. Darriba D, Taboada GL, Doallo R, Posada D. jModelTest 2: more models, new heuristics and parallel computing. Nat Methods. 2012;9:772. PubMed https://doi.org/10.1038/nmeth.2109

Appendix Table 1. Summary of Australian Ross River virus sequences used in the study*

Strain	Source†	Year collected	Location‡	GenBank accession no.
T48	Mosquito (*Aedes vigilax*)	1959	Townsville, QLD	GQ433359
2982	Bird (*Microeca fascinans*)	1965	Northeast Australia, QLD	GQ433355
3078	Bird (*Poephila personata*)	1965	Northeast Australia, QLD	GQ433356
8961	Marsupial (*Macropus agilis*)	1965	Northeast Australia, QLD	GQ433357
2975	Bird (*Grallina cyanoleuca*)	1965	Northeast Australia, QLD	GQ433360
9057	Marsupial (*Wallabia agilis*)	1968	Northeast Australia, QLD	GQ433358
NB5092	Mosquito (unspecified)	1969	Nelson Bay, NSW	M20162
Cairns90	Human	1990	Cairns, QLD	KX757000
Cairns91a	Human	1991	Cairns, QLD	KX756998
BNE91	Human	1991	Brisbane, QLD	KX757002
Cairns91b	Human	1991	Cairns, QLD	KX757003
RHCTN91	Human	1991	Rockhampton, QLD	KX757004
TSV91	Human	1991	Townsville, QLD	KX757006
NABR92	Human	1992	Nambour, QLD	KX757005
RDCF93	Human	1993	Redcliffe, QLD	KX756999
TSV94	Human	1994	Townsville, QLD	KX756997
B94–20	Mosquito (*Culex annulirostris*)	1994	Brisbane, QLD	KX757016
DC5692	Mosquito (*Aedes camptorhynchus*)	1995	Peel region, WA	HM234643
BNE96	Human	1996	Brisbane, QLD	KX757001
352–96	Mosquito (unspecified)	1996	Cairns, QLD	KX757009
211–97	Mosquito (unspecified)	1997	Cairns, QLD	KX757010
388A-98	Mosquito (unspecified)	1998	Cairns, QLD	KX757011
RCHTN00	Mosquito (*Mansonia uniformis*)	2000	Rockhampton, QLD	KX757007
QML1	Human	2004	Northeast Australia, QLD	GQ433354
71981–05	Mosquito (unspecified)	2005	Port Stephens, NSW	KX761985
SV64	Mosquito (*Verrallina carmenti*)	2007	Cairns, QLD	KX757008
LGRH-7021	Mosquito (FTA card)	2013	Longreach, QLD	KX757015
BNE2015a	Human	2015	Brisbane, QLD	KX757012
BNE2015b	Human	2015	Brisbane, QLD	KX757013
BNE-2885	Mosquito (FTA card)	2015	Brisbane, QLD	KX757014
188448	Mosquito (*Ae. procax*)	2015	Hawkesbury, NSW	KY290880
188449	Mosquito (*Ae. procax*)	2015	Hawkesbury, NSW	KY290881
188450	Mosquito (*Ae.* Marks sp. No. 51)	2015	Hawkesbury, NSW	KY290882
19661	Mosquito (FTA card)	2015	Tweed, NSW	KY290883
203412	Mosquito (*Cx. annulirostris*)	2015	Griffith, NSW	KY290884
203769	Mosquito (*Anopheles annulipes*)	2015	Leeton, NSW	KY290885
19775	Human	2016	Cairns, QLD	KY290875
19776	Human	2016	Cairns, QLD	KY290876
19777	Human	2016	Cairns, QLD	KY290877
19779	Human	2016	Townsville, QLD	KY290878
19780	Human	2016	Sunshine Coast, QLD	KY290879

*FTA, Flinders Technology Associates; NSW, New South Wales; QLD, Queensland; WA, Western Australia.
†Mosquito species of origin or FTA card source is in parentheses where information was available.
‡Locations of sample collections from QLD, NSW, and WA.

Appendix Table 2. Ross River virus-specific oligonucleotide primers used to amplify and sequence the E3 and E2 genes*

Primer†	Sequence	Genome position‡
8183for	5′-CAGCGGAGGAAGGTTTACCA-3′	8183–8202
8313for	5′-CTGTCTGTGGTGACGTGGAC-3′	8313–8332
8566for	5′-GCCGTAGTGTAACAGAGCAC-3′	8566–8585
8585rev	5′-GTGCTCTGTTACACTACGGC-3′	8585–8566
8870for	5′-CATCGTCGCACATTGTCCGC-3′	8870–8889
8999for	5′-TAGACCCCACTTTGGCGTAG-3′	8999–9018
9253rev	5′-GCAGCATGGCATTGGTCAAT-3′	9253–9234
9588for	5-CAACTGACGACCGAGGGCAAAC-3′	9588–9609
9609rev	5′-GTTTGCCCTCGGTCGTCAGTTG-3′	9609–9588
10245rev	5′-GGTAGTCTGGCTGCTCCTTG-3′	10245–10226
10298rev	5′-ACAGTAGGCTCCACCCCACA-3′	10298–10279

*E, envelope; for, forward; rev, reverse.
†Sense and antisense primers are labeled as for or rev.
‡Sequence positions are based on the Ross River virus T48 sequence, GenBank accession number DQ226993.

Appendix Table 3. Total numbers of mosquitoes collected across the 9 sites used in this study by species and year*

Species	2011–2012	2012–2013	2013–2014	2014–2015
Aedes aculeatus	1,343	1,075	63	911
Ae. alboannulatus	1	0	0	3
Ae. alboscutellatus	0	9	1	10
Ae. alternans	41	347	195	647
Ae. bancroftianus	0	0	0	1
Ae. burpengaryensis	886	353	0	206
Ae. gahnicola	1	1	3	0
Ae. lineatopennis	523	162	30	1,244
Ae. mallochi	0	1	0	0
Ae. notoscriptus	892	1,610	2,210	2,899
Ae. procax	5,096	4,654	1,570	26,408
Ae. vigilax	18,580	123,024	84,133	211,008
Ae. vittiger	385	230	163	2,068
Ae. wasselli	0	1	0	0
Anopheles annulipes	823	1,189	992	1,036
An. atratipes	64	94	44	43
An. bancroftii	33	5	3	59
Coquillettidia linealis	10,904	19,403	1,666	2,830
Cq. xanthogaster	736	75	30	128
Culex annulirostris	31,451	39,858	12,650	140,287
Cx. australicus	0	0	13	14
Cx. bitaeniorhynchus	33	11	2	8
Cx. cyclindricus	0	0	0	2
Cx. globocoxitis	0	0	0	1
Cx. orbostiensis	3,779	3,591	2,164	6,748
Cx. quinquefasciatus	8	0	18	49
Cx. sitiens	223	539	549	2,595
Mansonia uniformis	520	1,651	334	2,800
Mimomyia elegans	1	0	0	3
Mi. metallica	1	0	0	0
Tripteroides punctolateralis	1	0	0	0
Uranotaenia nivipes	2	6	2	9
Ur. pygmaea	1	0	0	1
Verrallina funerea	1,050	6,153	1,202	1,810
Ve. Marks sp. no. 52	20	169	364	1,426
Undetermined	106	9	21	6,074
TOTAL	77,504	204,220	108,422	411,328

*Traps were operated weekly at sites across the Brisbane local government area.

Appendix Table 4. Mosquitoes collected from 9 sites in Brisbane, Australia, in 2015, and processed for virus detection*

Mosquito species	Total number processed	Pools tested by CC-ELISA		Pools tested by rRT-PCR	
		Number tested	Number positive	Number tested	Number positive
Aedes aculeatus	196	9	0	5	0
Ae. alboannulatus	1	1	0	0	0
Ae. alternans	68	7	0	1	0
Ae. burpengaryensis	40	5	0	1	0
Ae. lineatopennis	339	12	0	6	0
Ae. notoscriptus	65	6	0	1	0
Ae. procax	2,303	37	0	9	0
Ae. vigilax	4,604	60	0	10	1
Ae. vittiger	220	15	0	4	0
Anopheles annulipes	25	9	0	4	0
An. bancroftii	37	6	0	4	0
Coquillettidia linealis	77	5	0	1	0
Cq. xanthogaster	19	7	0	4	0
Culex annulirostris	9,413	107	1	53	1
Cx. orbostiensis	91	13	0	3	1
Cx. pullus	5	1	0	0	0
Cx. sitiens	53	5	0	1	0
Mansonia uniformis	773	21	0	12	1
Verrallina funerea	98	14	0	4	0
Ve. Marks sp. No. 52	277	18	0	6	0
Damaged specimens†	2,422	27	0	26	11
TOTAL	21,250	385	1	155	15

*CC-ELISA, cell culture ELISA; rRT-PCR, real-time reverse transcription PCR.

†Mosquitoes were damaged by rain which had permeated the trap collection and precluded morphological identification.
